# Development and Performance Evaluation of Geopolymer-Based Fluidized Solidified Soil Using Phosphogypsum and Slag Powder for Road Backfilling

**DOI:** 10.3390/ma18235256

**Published:** 2025-11-21

**Authors:** Xiaojuan Li, Ping Zheng, Honglei Lu, Shiyu Zhu, Haochen Tian, Xiaoping Ji

**Affiliations:** 1 Shaanxi Transportation Holding Group Technology Development Co., Ltd., Xi’an 710075, China; lxiaojuan2024@163.com; 2 Xinjiang Key Laboratory for Safety and Health of Transportation Infrastructure in Alpine and High-Altitude Mountainous Areas, Xinjiang Transportation Planning Survey and Design Institute Co., Ltd., Urumqi 830006, China; 3 Department of Automotive Engineering, Guizhou Aerospace Vocational and Technical College, Zunyi 563000, China; luhonglei523@163.com; 4 School of Highway, Chang’an University, Xi’an 710064, China; tianhaochen0828@163.com (H.T.); jixp82@163.com (X.J.); 5 School of Civil Engineering, Anhui Jianzhu University, Hefei 230009, China

**Keywords:** industrial solid waste, backfilling engineering, phosphogypsum-based geopolymer, fluidized solidified soil, mechanical properties, leaching of toxic substances

## Abstract

The large-scale and high-value utilization of industrial solid waste has become a key research area in sustainable building materials. However, ensuring effective backfilling quality in narrow or irregular spaces remains challenging in civil engineering. Developing flowable solidification materials from industrial solid waste not only resolves issues inherent in traditional backfilling techniques but also enhances efficient resource utilization. In this study, phosphogypsum was used to prepare geopolymers, which served as binders replacing cement in producing phosphogypsum-based fluidized solidified soil (PFSS). The workability, mechanical strength, and toxic substance leaching of PFSS were evaluated. Moreover, the underlying mechanisms of strength formation and toxic substance immobilization were investigated. The optimal PFSS composition was determined to have a water-to-solid ratio of 0.48–0.50 and a geopolymer content of 12–18% (by mass). Under these conditions, the material exhibited fluidity ranging from 160 to 220 mm, a 28-day compressive strength of 0.86 MPa, a California Bearing Ratio (CBR) of 8%, and a resilient modulus of 40 MPa. These parameters satisfy the performance standards required for backfilling in high-grade highways. The leaching concentrations of heavy metals (As, Pb, and Cr) complied with China’s Class III groundwater quality standards. Microstructural analyses indicated the occurrence of hydration, pozzolanic reactions, geopolymerization, and carbonation. Microstructural analyses indicated the formation of an interlocking three-dimensional network, composed of C-S-H, C-A-S-H gels, and ettringite (AFt), which contributes significantly to the strength development and immobilization of heavy metals. These products collectively formed an interlocking three-dimensional network structure, significantly contributing to PFSS strength development. Heavy metals were effectively immobilized within the matrix due to the combined effects of physical adsorption and chemical bonding.

## 1. Introduction

Phosphogypsum, an acidic and hazardous industrial by-product of phosphoric acid production, poses a significant environmental challenge [1,2]. The global stockpile of phosphogypsum currently amounts to 6 × 10^9^ tons, with an annual increase of approximately 1.5 × 10^8^ tons. While some countries repurpose phosphogypsum for applications such as the production of cooked gypsum powder and gypsum-based building materials, others resort to disposal in landfills or marine environments [3]. China, the world’s largest producer of phosphate fertilizers, has accumulated around 5 × 10^8^ tons of phosphogypsum. Due to its inherent characteristics, a substantial portion is stored in large landfills or rivers, with about 85% remaining unused. Therefore, it is critical to achieve rational and efficient utilization of phosphogypsum [4].

Various methods have been explored for reusing phosphogypsum, including its use as a cement retardant, construction gypsum, and soil modifier [5,6] demonstrated that modifying phosphogypsum with fly ash and calcium carbide slag significantly delays cement setting times. Calderón-Morales et al. (2021) [7] found that phosphogypsum could be recycled into construction gypsum, achieving impressive compressive strength. Guanru Lu et al. (2023) [8] showed that applying phosphogypsum to alkaline soils markedly improves soil quality. However, the environmental pollution and radiation risks associated with phosphogypsum remain a concern [9]. Developing a new cementitious material from phosphogypsum could provide a sustainable solution for its large-scale accumulation [10]. Researchers have also explored blending phosphogypsum with other cementitious materials for road sub-base applications. Değirmenci (2008) [11] studied the calcination of phosphogypsum and blended it with lime and coal fly ash. This approach achieved a 28-day compressive strength of 13.76 MPa with 50% incorporation of calcined phosphogypsum. Ngo et al. (2022) [12] mixed phosphogypsum, cement, and sand in different ratios. A mixture ratio of 7:1:2 resulted in a 28-day compressive strength of 1.38 MPa. Liang (2023) [13] found that mixing phosphogypsum with lime and cement at phosphogypsum dosages between 40% and 45% achieved a 7-day unconfined compressive strength exceeding 2.0 MPa. This met the compressive strength requirements for highway subgrades. Although these approaches have increased phosphogypsum utilization, they have not adequately addressed the issue of toxic substance leaching.

Geopolymers are cementitious materials formed through the reaction of silica-aluminate substances when exposed to alkali activation [14]. Previous studies have shown that phosphogypsum and silica-aluminate substances can hydrate and form dense structures under alkali excitation, thereby enhancing strength and immobilizing toxic substances. Oubaha et al. (2024) [15] utilized sodium hydroxide (NaOH) as an alkali activator to produce reactive materials by combining phosphogypsum with clay. With a phosphogypsum dosage of 22.4%, the compressive strength of the specimen reached 27.3 MPa, making it suitable for use in road base layers. Pratap et al. (2024) [16] employed phosphogypsum, bauxite residues (BR), and fly ash (FA) as raw materials. These were blended in a 6:2:2 ratio to create geopolymer concrete, using NaOH and sodium silicate as alkali activators. The maximum compressive strength achieved was 54.74 MPa. Zhang et al. (2023) [17] developed geopolymers using phosphogypsum, fly ash mixed with slag as raw materials, and lime as an alkali activator. The geopolymer mortar achieved a compressive strength of 55.5 MPa, using phosphogypsum, fly ash, slag, and lime in proportions of 40%, 20%, 40%, and 2%, respectively. These studies underscore the potential application of phosphogypsum-based geopolymers in road sub-base and base filler applications. However, some limitations persist. First, none of the studies utilized phosphogypsum as a primary raw material for geopolymer preparation. Second, there is a lack of targeted research on the specific road layers where these materials can be effectively applied. This limits the large-scale and high-quality utilization of phosphogypsum in road engineering. Addressing these gaps could facilitate a more comprehensive and efficient utilization of phosphogypsum in road infrastructure.

Ensuring the quality of backfilling in narrow or irregular spaces, such as bridge approaches, has long been a challenge in civil engineering. Improper compaction during construction can easily damage embedded structures, severely affecting quality. The adoption of self-flowing pouring technology is considered an effective measure for filling and reinforcing such backfilling projects [18]. Utilizing industrial solid waste to prepare fluidized solidified soil for these backfilling applications can resolve a range of issues associated with traditional backfilling methods while promoting resource utilization of industrial waste. Currently, phosphogypsum is primarily used in road base layers; however, it falls short in terms of workability. While cement-based, fluid-cured soil meets the necessary requirements, it poses significant environmental risks. The production of every ton of cement results in approximately 0.8 tons of carbon dioxide (CO_2_) emission [19]. However, these previous approaches primarily focused on producing stiff mixtures for base or sub-base layers, overlooking the critical workability requirements (e.g., fluidity, setting time) essential for fluidized solidified soil used in challenging backfilling scenarios. Furthermore, while some studies utilized phosphogypsum as a supplementary component, none employed it as the primary raw material for geopolymer preparation specifically designed for fluidized applications, limiting its high-value and large-scale utilization.

This study focuses on using phosphogypsum to create geopolymers as a cement alternative for fluid-cured soil preparation. Initially, the process for creating geopolymers from phosphogypsum was optimized. Subsequently, fluid-cured soil was developed using the geopolymer as a cementitious material. Laboratory tests were conducted to evaluate the mechanical properties of fluidized solidified soil, confirming its suitability for challenging backfilling applications such as road transition sections, retaining walls, and bridge abutments. The study also explored the mechanisms of strength formation and the solidification of toxic substances using microscopic methods. The findings highlight the potential for large-scale, high-quality, and environmentally friendly utilization of phosphogypsum in road engineering, contributing to resource conservation and environmental protection.

## 2. Materials and Methods

### 2.1. Raw Materials

#### 2.1.1. Phosphogypsum

**Figure 1:**
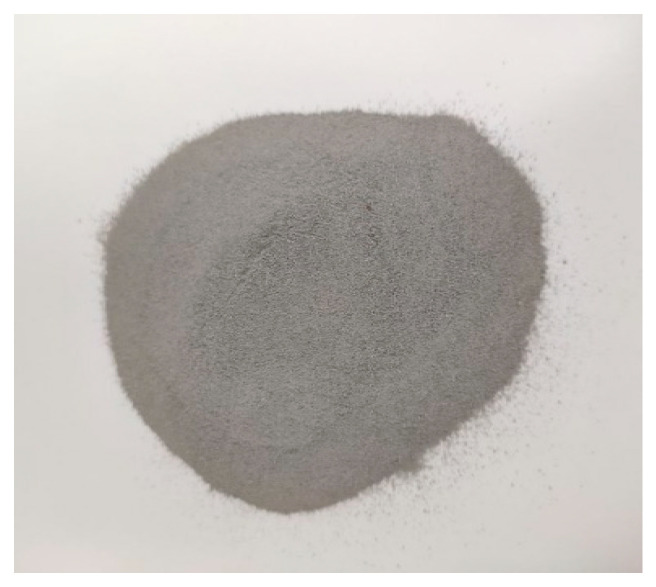
Phosphogypsum for experaiments.

**Figure 2:**
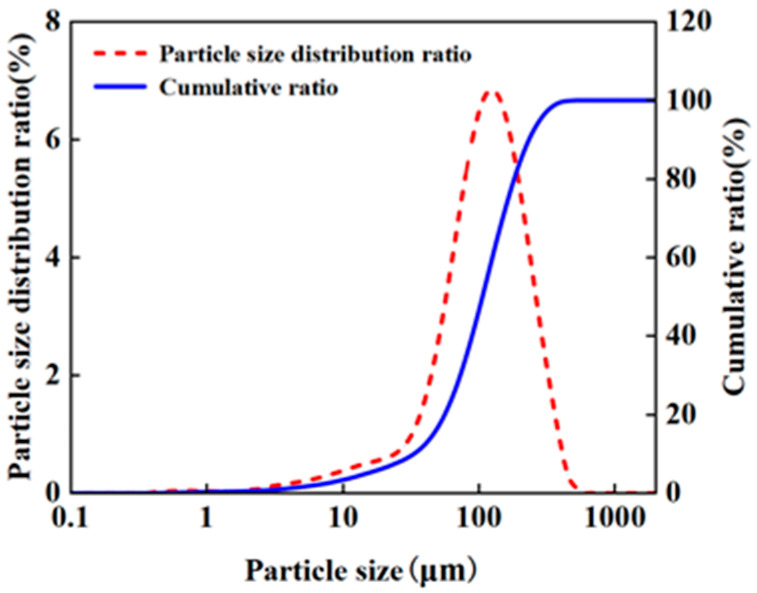
Particle size distribution of phosphogypsum.

The phosphogypsum used in the experiments was sourced from the Panlong Slag Yard, Mianzhu County, Deyang City, Sichuan Province, China. After high-temperature drying, the phosphogypsum appeared as a grayish-white powder, as shown in Figure 1. The particle size, chemical composition, and leaching concentration of toxic substances were analyzed. The results are presented in Figure 2, Table 1 and Table 2, respectively.

The particle size of the samples ranged from 0.1 to 600 μm, with an average particle size of 122.2 μm. The contents of CaO and SO_3_ in the phosphogypsum were 49.57% and 38.74%, respectively. However, the SiO_2_ and Al_2_O_3_ contents were relatively low at 4.22% and 1.86%, respectively, limiting the effectiveness of geopolymerization. The concentrations of Pb, Cr, and F^−^ were found to be 101.85 μg/L, 682.50 μg/L, and 14.64 μg/L, respectively. These values exceeded the Class IV groundwater quality standards specified in “Standards for Groundwater Quality” (GB/T14848-2017 [20]), which limit these substances to 100, 100, and 2 μg/L, respectively.

SiO_2_ and Al_2_O_3_ are the primary active substances involved in geopolymerization reactions, as they are capable of forming strength under alkali activation. Due to the low content of SiO_2_ and Al_2_O_3_ in phosphogypsum, it is necessary to incorporate an admixture rich in silica-alumina components. In this study, slag powder was selected as an additive, with a total SiO_2_ and Al_2_O_3_ content of 41.55%, as shown in Table 3.

#### 2.1.2. Alkali Exciters

The geopolymerization reaction requires an alkaline environment. In this study, NaOH and Na_2_SiO_3_ were selected as alkali activators. These two materials were combined to form a composite alkali activator, which overcomes the limitations of using a single alkali activator [21]. The composite alkali activator provides an alkaline environment and Na^+^ metal ions to promote the initial geopolymerization reaction and supply the SiO_2_ needed for the subsequent hydration process. The composite alkali activator was prepared by mixing sodium hydroxide (NaOH) solution with sodium silicate (Na_2_SiO_3_) solution. The NaOH solution with a concentration of 8 mol/L was prepared by dissolving NaOH pellets (≥96% purity) in deionized water. The modulus (SiO_2_/Na_2_O molar ratio) of the composite alkali activator was adjusted to 1.4 by controlling the mixing ratio of Na_2_SiO_3_ and NaOH solutions.

#### 2.1.3. Soil

Clay was selected for the preparation of geopolymer fluidized stabilized soil (PFSS). Clay is rich in substantial amounts of Al_2_O_3_ and SiO_2_, which can promote the geopolymerization reaction with phosphogypsum. The physical properties of the clay are presented in Table 4. Based on the results, it can be concluded that clay has a low liquid limit.

### 2.2. Test Methods

#### 2.2.1. Workability Tests

**Figure 3:**
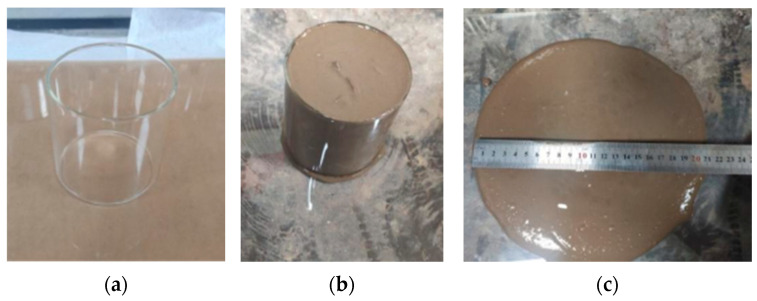
Fluidity test: (**a**) Hollow glass cylinders; (**b**) pouring of test cylinders; (**c**) extensibility measurement.

Workability includes flowability, setting time, and bleeding rate. It plays a critical role in determining whether fluidized stabilized soil (PFSS) can be effectively applied in construction. Flowability is a significant indicator used to characterize the workability of PFSS. Smooth, hollow Plexiglas cylinders with a height and diameter of 80 mm were used to measure flowability through the extensibility method. This method follows the study by Lee et al. [22] and the “Standard for Test Method of Performance on Ordinary Fresh Concrete” (GB/T 50080-2002 [23]). The test procedure is shown in Figure 3. The setting time of PFSS is crucial for construction. The initial and final setting times were determined using the Vickers meter method, according to the “Standard for Geotechnical Testing Methods” (GB/T 50123-2019). The bleeding rate reflects the stability of PFSS. A graduated glass measuring cup was utilized to determine the 2 h bleeding rate of PFSS. Equation (1) was used to calculate the bleeding rate, following the “Test Methods of Cement and Concrete for Highway Engineering” (JTG 3420-2020 [24]). Specifically, the initial height of the freshly poured slurry (*H*_1_) and the final height of the soil paste after water segregation (*H*_2_) were directly measured using the graduated glass measuring cup at the prescribed time. The bleeding rate was then calculated as the percentage decrease in height relative to the initial height.

(1) $$Bleeding\;rate=\frac{H_{1}-H_{2}}{H_{1}}\times 100\%,$$

where *H*_1_—Surface height of just-poured geopolymer fluid-cured soil slurry; and *H*_2_—Surface height of geopolymer fluid-cured soil curing body after segregation.

#### 2.2.2. Mechanical Strength Test

**Figure 4:**
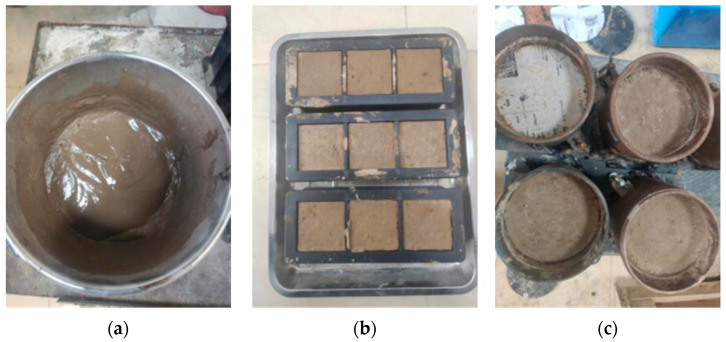
PFSS specimen preparation: (**a**) PFSS slurry; (**b**) cubic specimen; (**c**) CBR specimen.

**Figure 5:**
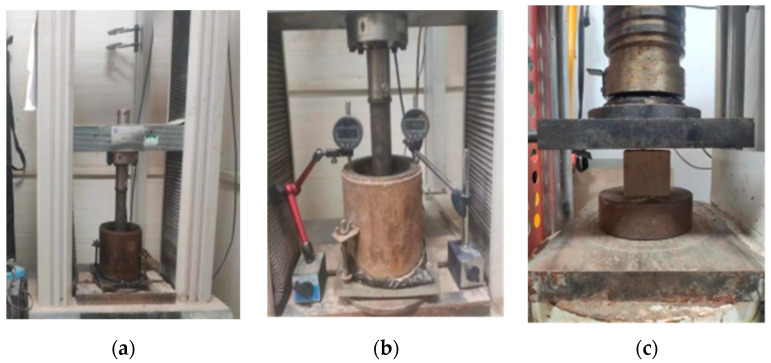
Mechanical tests of PFSS: (**a**) CBR test; (**b**) compressive resilient modulus test; (**c**) compressive strength test.

This section tests the compressive strength, CBR value and compressive resilient modulus of the specimens. Cuboid specimens with an edge length of 70.7 mm were fabricated to test the unconfined compressive strength, and cylinder specimens with a height of 170 mm and a diameter of 152 mm to test the CBR value and compressive resilient modulus, referring to “Test Methods of Soils for Highway Engineering” (JTG 3430-2020 [25]). The specimens were cured at a temperature of 40 ± 2 °C and a humidity of 95%, with curing ages of 3, 7, 14 and 28 days, respectively. Specimen preparation is shown in Figure 4 and specimen test is shown in Figure 5.

#### 2.2.3. Toxic Substance Leaching Tests

**Figure 6:**
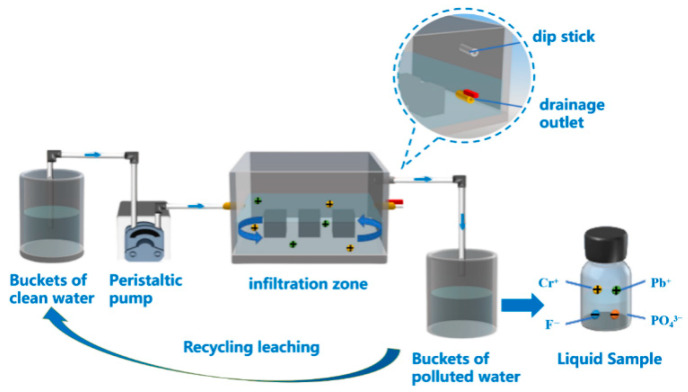
Toxic ion circulating leaching device (adapted from Ji et al., 2023 [26]).

To investigate the leaching concentration of hazardous ions when PFSS is exposed to groundwater in road subgrades, a recirculating leaching device is presented in this paper [26], as shown in Figure 6. The leaching device unit mainly consists of a bucket of clean water, a peristaltic pump, an infiltration zone, and a bucket of polluted water. First, the specimens were placed into the infiltration zone with a spacing greater than 10 mm. After placement, distilled water was added until the liquid level reached the overflow outlet. During the experiment, the peristaltic pump was turned on, allowing distilled water to flow into the bottom of the infiltration zone. The infiltration replenishment rate was set to 50 mL/min. When the water level in the infiltration zone exceeded the outlet, the liquid infiltrating through the specimens flowed into the polluted water bucket through a water pipe. After the distilled water from the clean-water bucket was completely transferred, the water collected in the polluted-water bucket was returned to the clean-water bucket. The above steps were then repeated. Finally, inductively coupled plasma (ICP) analysis was used to determine the concentration of toxic ions in the leaching solution according to the “Standard for Groundwater Quality” (GB/T 14848-2017) [20].

#### 2.2.4. Microscopic Test

Scanning electron microscopy (SEM) was used to analyze the micro-morphology of PFSS. The mechanism of strength formation and toxic substance solidification in PFSS were also explored.

### 2.3. Preparation and Performance of PFSS

**Figure 7:**
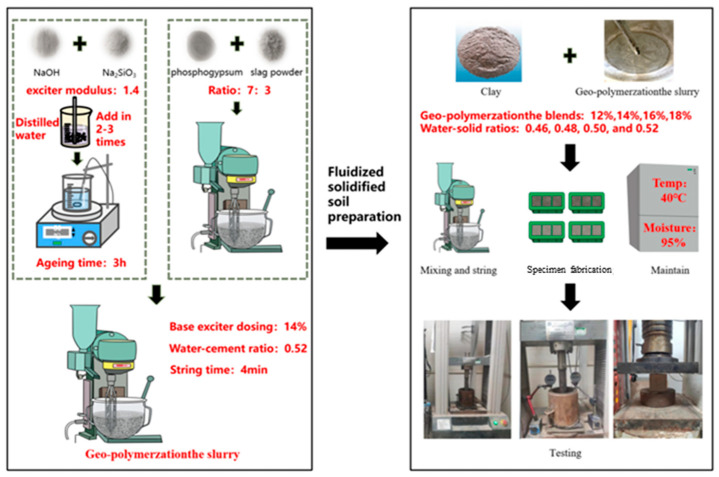
General flow of PFSS preparation.

Phosphogypsum was used to prepare geopolymers. The phosphogypsum-based geopolymer (PBG) was then utilized as binding materials for PFSS preparation. Geopolymers meeting the strength requirements were selected and mixed with clay. Referring to previous studies by our research group [26], specimens were prepared with different geopolymer blends and water-to-solid ratios to investigate the properties of fluid-cured clays, and the detailed preparation process is illustrated in Figure 7.

There are four influencing factors for the preparation: A (phosphogypsum: slag powder ratio), B (alkali activator modulus), C (alkali activator dosage), and D (water-to-solid ratio). Referring to related studies [16], values of A = 7:3, B = 1.4, C = 14%, and D = 0.52 were selected for the present study. The preparation involved an aging time of 3 h, stirring time of 4 min, and curing temperature of 30 °C.

**Figure 8:**
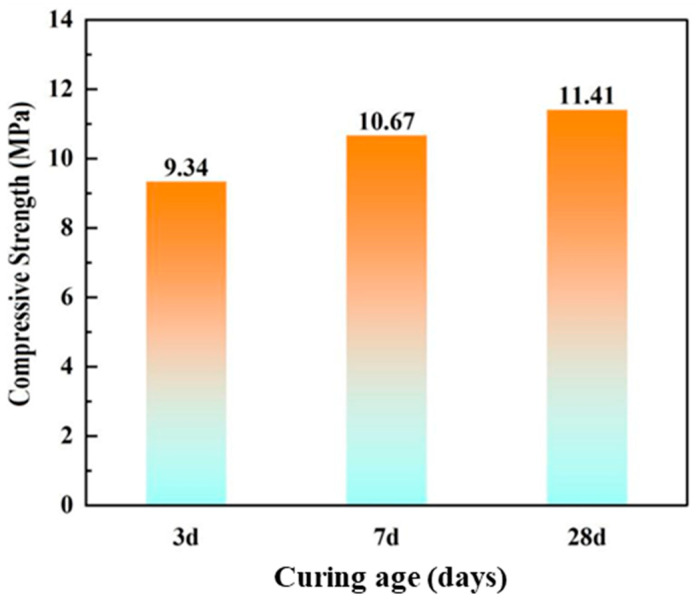
Compressive strength of phosphogypsum base geopolymer.

The phosphogypsum-based geopolymer ratios and the unconfined compressive strengths of the test specimens are presented in Table 5 and Figure 8. According to the “Technical Guidelines for Construction of Highway Roadbases” (JTG/T F20-2015) [27], the prepared geopolymers meet the strength requirements for use as binding materials in road backfill, as shown in Table 6. Low-liquid-limit clay was mixed with geopolymer to formulate fluid stabilized soil. Geopolymer dosages of 12%, 14%, 16%, and 18%, combined with water-to-solid ratios of 0.46, 0.48, 0.50, and 0.52, were selected to prepare PFSS specimens and evaluate their performance.

## 3. Results and Discussion

### 3.1. Workability

#### 3.1.1. Fluidity

**Figure 9:**
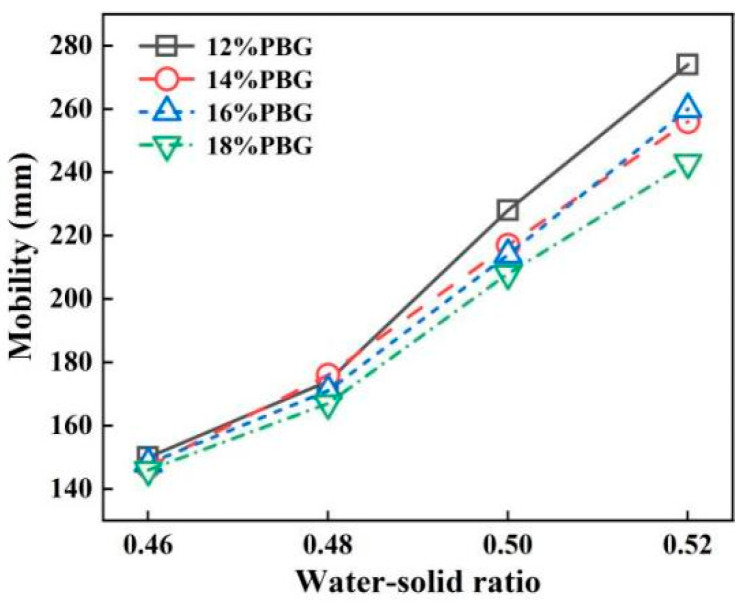
Changing pattern of fluidity with water–solid ratio.

**Figure 10:**
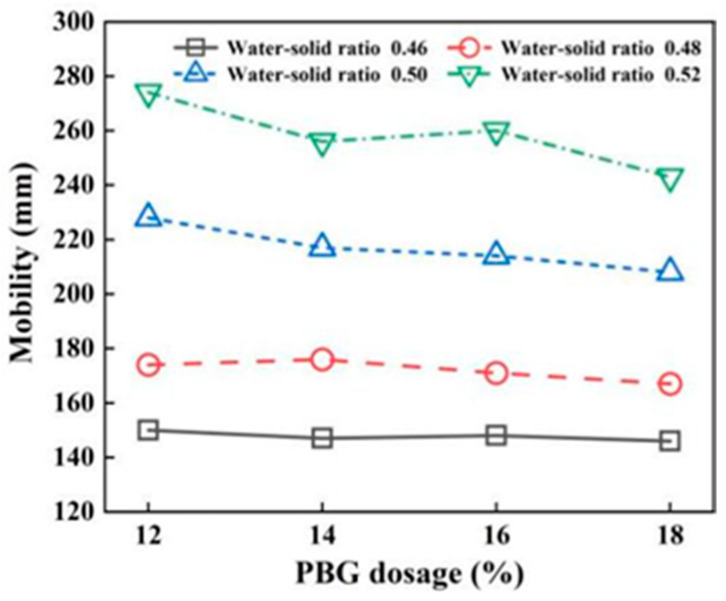
Changing pattern of fluidity with geopolymer dosage.

The geopolymer dosages of 12, 14, 16, and 18%, along with water–solid ratios of 0.46, 0.48, 0.50, and 0.52, were selected for the fluidity tests. The results are presented in Figure 9 and Figure 10. From the findings, it can be concluded that fluidity increases with an increasing water–solid ratio. The effect of geopolymer dosage on flowability is not significant. This increase in fluidity is attributed to the higher water content within the slurry, resulting from the elevated water–solid ratio.

When the water–solid ratio is 0.48~0.50, the fluidity of PFSS meets the requirements of 160~220 mm. Specified by “Technical standard for engineering application by using premixed fluidized stabilized soil” (DBJ51/T 188-2022) [28]. When the water–solid ratio is 0.50, the fluidity of PFSS meets the requirement of >220mm.

#### 3.1.2. Solidification Times

**Figure 11:**
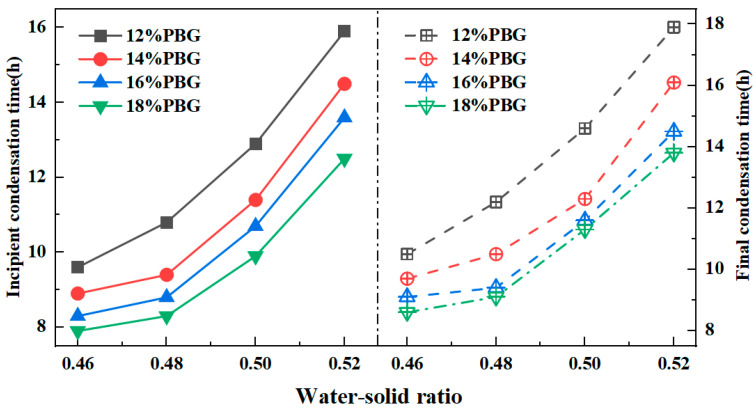
Variation rule of solidification time with water–solid ratio.

**Figure 12:**
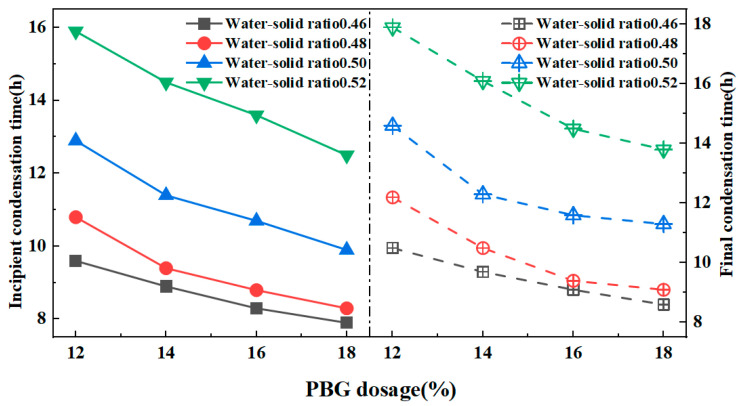
Variation rule of coagulation time with geopolymer dosage.

The patterns of solidification times are illustrated in Figure 11 and Figure 12. The results indicate that the solidification times of PFSS increased as the water-to-solid ratio rose. Conversely, an increase in geopolymer dosage led to a reduction in the setting time of the PFSS. This effect can be attributed to the higher water-to-solid ratio, which lowers the ionic concentration in the geopolymer reaction system due to excess free water in the slurry. This consequently slows down the reaction rate. Based on the results, when the water-to-solid ratio ranged from 0.46 to 0.52, the setting times of PFSS satisfied the relevant specification requirements (initial setting time ≥ 3 h, final setting time ≤ 24 h), as stipulated in the “Technical Standard for Engineering Application by Using Premixed Fluidized Stabilized Soil” (DBJ51/T 188-2022) [28].

#### 3.1.3. Bleeding Rate

**Figure 13:**
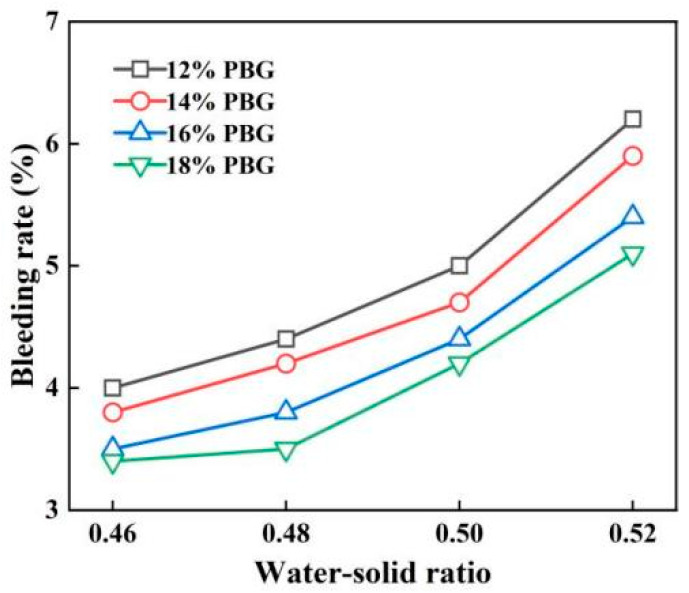
Variation pattern of water secretion rate with water–solid ratio.

**Figure 14:**
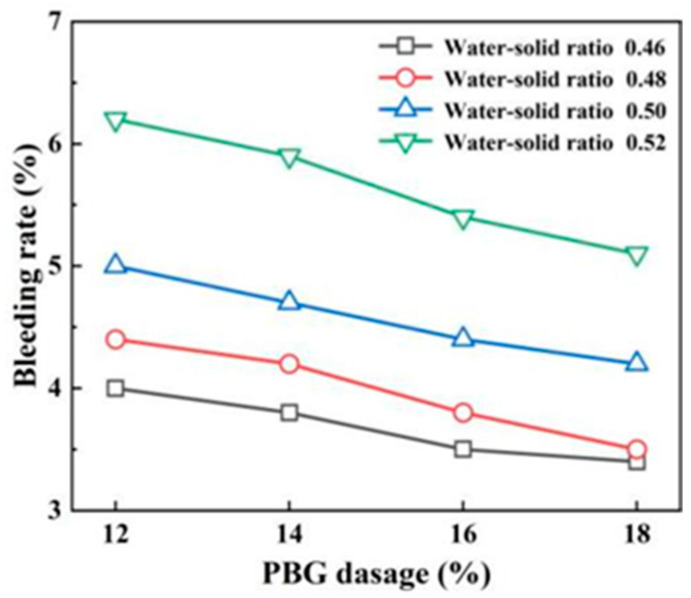
Variation pattern of water secretion rate with geopolymer mixing amount.

The variations in bleeding rate are illustrated in Figure 13 and Figure 14. The results indicate that the bleeding rate increases with a higher water-to-solid ratio and decreases with an increased geopolymer dosage. Specifically, as the water-to-solid ratio rises, the amount of free water also increases. Any excess water will then seep out once the soil particles have absorbed sufficient water to reach saturation. Conversely, with a higher geopolymer dosage, the phosphogypsum and slag powder have a significantly smaller specific surface area than soil particles, leading to higher water absorption. This consequently reduces the bleeding rate. When the water-to-solid ratio ranges from 0.46 to 0.50, the bleeding rates of PFSS at different geopolymer dosages remain below 5%, thus meeting the requirements outlined in the “Technical Standard for Engineering Application by Using Premixed Fluidized Stabilized Soil” (DBJ51/T 188-2022) [28].

As a result of the experiments conducted, a water–solid ratio of 0.48 was selected, as it effectively met the engineering requirements. In the following section, specimens were prepared using varying dosages of geopolymer to determine the optimum ratio.

### 3.2. Mechanical Strength

In this section, the dosages of geopolymer were selected at 12%, 14%, 16%, and 18%, with a water-to-solid ratio of 0.48 for the mechanical strength tests. The dosage of geopolymer that satisfied the strength requirements was determined based on the experimental data.

#### 3.2.1. California Bearing Ratio (CBR)

**Figure 15:**
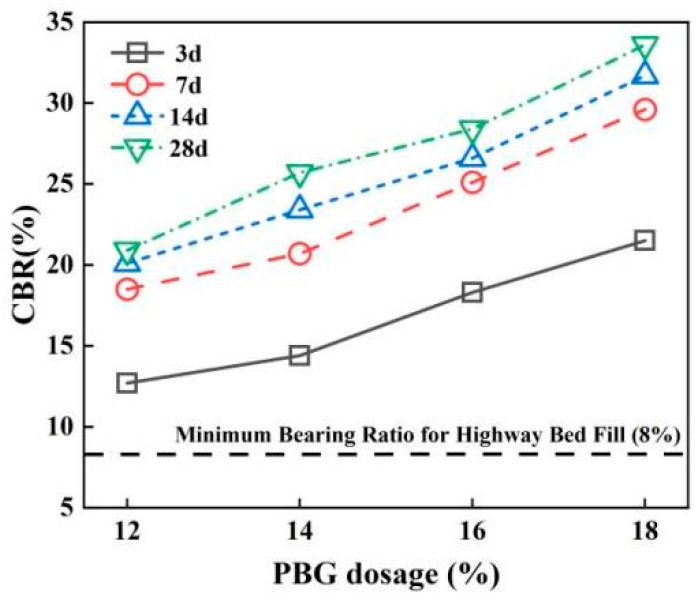
Effect of different geopolymer dosages on CBR.

**Figure 16:**
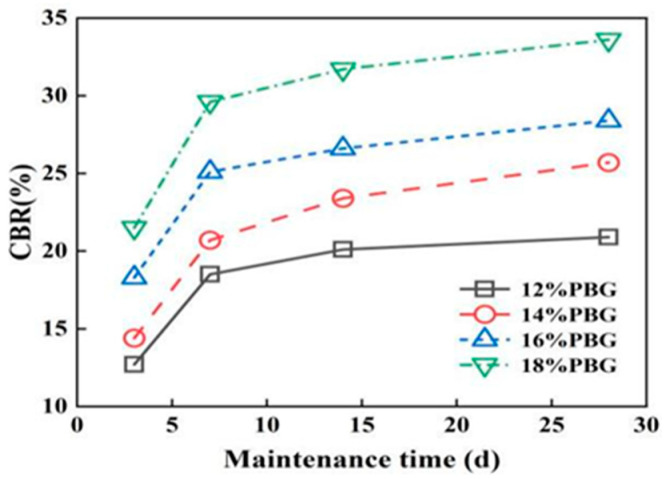
Effect of different maintenance times on CBR.

The effects of geopolymer dosage and curing time on CBR values are illustrated in Figure 15 and Figure 16. The results indicate that the CBR of PFSS improves with an increase in geopolymer dosage. This enhancement is attributed to the increased geopolymer dosage, which promotes the formation of hydrated gel products. These products densify the soil structure and consequently enhance the bearing capacity. After 7 days of curing, the PFSS achieves 80% of its maximum bearing capacity. This meets the bearing capacity requirements for roadbeds designed for very heavy traffic loads, as specified in the “Specifications for Design of Highway Subgrades” (JTG D30-2004) [29].

#### 3.2.2. Resilience Modulus

**Figure 17:**
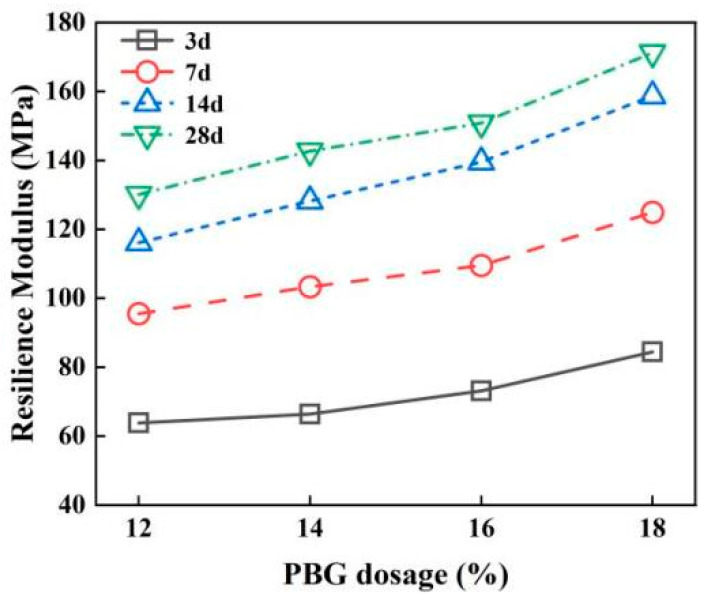
Effect of different geopolymer dosages on resilience modulus.

**Figure 18:**
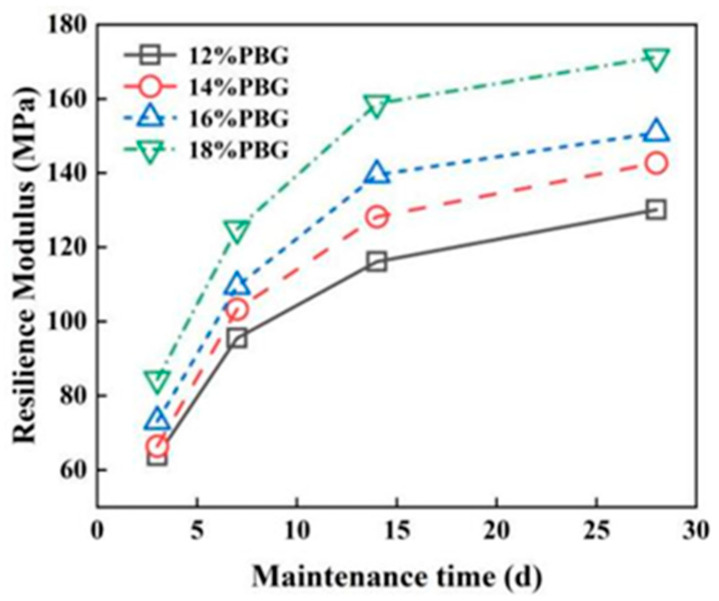
Effect of different maintenance ages on resilience modulus.

The effects of geopolymer dosage and curing time on resilience modulus are illustrated in Figure 17 and Figure 18. The results indicate that as the geopolymer dosage increases, the resilience modulus of the PFSS improves. Initially, the resilience modulus increases sharply; however, this trend gradually stabilizes as curing time progresses. The increased geopolymer dosage leads to the formation of more hydrated gel products, which fill the pores between soil particles and thereby enhance the resilience modulus of the PFSS. The rapid hydration rate observed in the early stages of the geopolymer reaction is attributed to the dissolution of Si and Al, which accelerates the reaction. As the active substances become depleted, the strength gain in later stages gradually slows down. According to the “Specifications for Design of Highway Subgrades” (JTG D30-2004) [29], the PFSS satisfies the requirement of a resilience modulus greater than 40 MPa at various curing ages. The resilience modulus measured in this study can be used as an indicator to characterize the stiffness of the material. Subsequent research will further analyze its stiffness parameters such as elastic modulus through a complete stress–strain curve [30,31].

#### 3.2.3. Unconfined Compressive Strength

**Figure 19:**
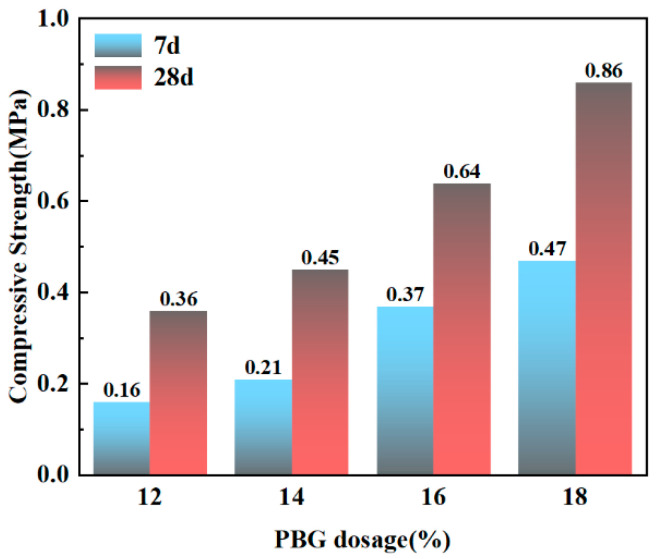
Compressive strength of PFSS.

The variation pattern of compressive strength in PFSS is illustrated in Figure 19. The compressive strength increases with higher geopolymer dosages and longer curing times. There are two primary reasons for this observation. First, as geopolymer dosage increases, the geopolymer reaction produces more hydration products and cementitious materials, thereby enhancing soil strength. Second, the geopolymer reaction proceeds more effectively over extended curing periods, generating additional cementitious materials and contributing to further strength development. The compressive strength can reach 0.86 MPa, which satisfies the strength requirement for backfill sections (28-day compressive strength ≥ 0.8 MPa) specified in the “Technical Standard for Engineering Application by Using Premixed Fluidized Stabilized Soil” (DBJ51/T 188-2022) [28].

It is noteworthy that the compressive strength of PFSS, while meeting the specification requirements for backfilling (≥0.8 MPa), is modest compared to some geopolymer-based materials reported in the literature. This is considered a deliberate trade-off in the mixed design. The high content of phosphogypsum, which has limited reactivity, and the relatively low alkalinity of the system were optimized to prioritize excellent workability (fluidity and setting time) and ensure effective immobilization of toxic substances, which are the primary goals for this specific application in flowable backfill [32,33].

### 3.3. Leaching Tests for Toxic Substances

**Figure 20:**
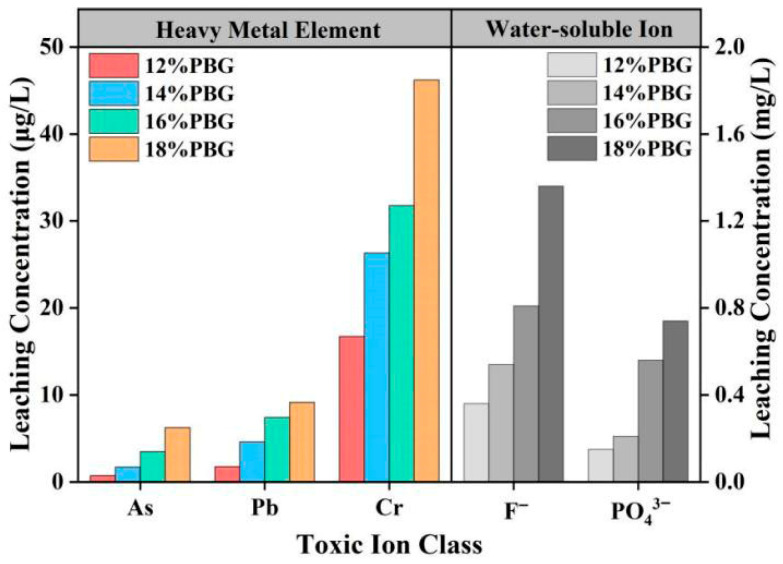
Toxic ion leaching concentrations of PFSS specimens.

In this section, specimens with a curing period of 7 days were selected to evaluate the leaching concentrations of toxic ions. The original phosphogypsum contained substantial amounts of water-soluble toxic ions, including F^−^ and PO_4_^3−^, as well as heavy metals such as As, Pb, and Cr. Consequently, it was essential to assess the environmental pollution risks associated with the use of PFSS as backfill material in road construction. The leaching concentrations of toxic ions are illustrated in Figure 20.

The results demonstrate that the toxic ion concentrations in the PFSS containing 12% geopolymer were significantly lower than those in the original phosphogypsum. According to the “Standard for Groundwater Quality” (GB/T 14848-2017) [20], the concentrations of As, Pb, and F^−^ in the leaching solution complied with Groundwater Quality Standard II, while the concentration of Cr met the criteria for Groundwater Quality Standard III. The leaching concentrations of As, Pb, Cr, F^−^, and PO_4_^3−^ ions from the 12% geopolymer-doped PFSS were 0.73, 1.76, 16.74 μg/L, 0.36, and 0.15 mg/L, respectively. Compared to the raw phosphogypsum, the leaching concentrations of As, Pb, Cr, F^−^, and PO_4_^3−^ in the PFSS with 12% geopolymer were reduced by approximately 96.6%, 98.3%, 97.5%, 97.5%, and 98.1%, respectively, demonstrating the effective solidification and immobilization of toxic substances. The effective immobilization of heavy metals and anions can be attributed to multiple mechanisms: (1) Precipitation of low-solubility hydroxides (e.g., Pb(OH)_2_) or complex salts under the alkaline condition; (2) physical encapsulation and adsorption by the formed C-A-S-H and C-S-H gels; and (3) chemical incorporation into the structure of ettringite (AFt) for certain anions [33,34,35].

### 3.4. Mechanisms of Strength Formation and Toxic Substances Solidification

**Figure 21:**
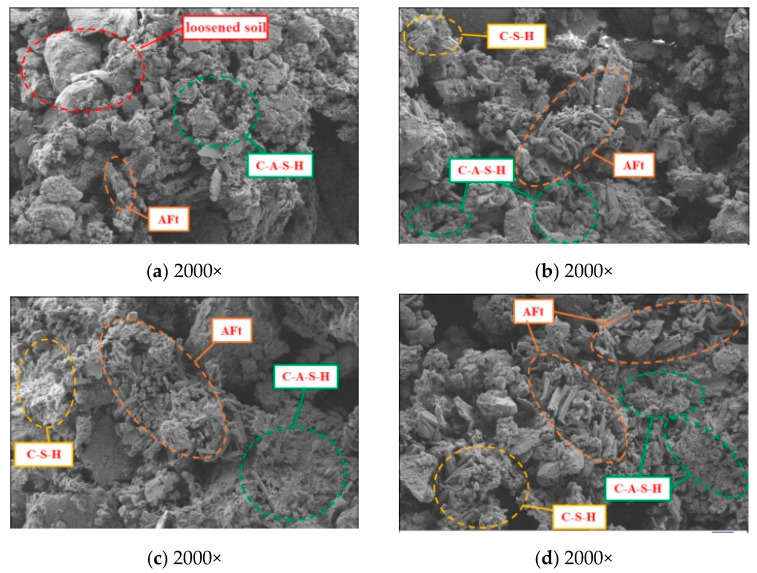
Microscopic morphology of PFSS (2000×): (**a**) 12% geopolymer content; (**b**) 14% geopolymer content; (**c**) 16% geopolymer content; (**d**) 18% geopolymer content.

**Figure 22:**
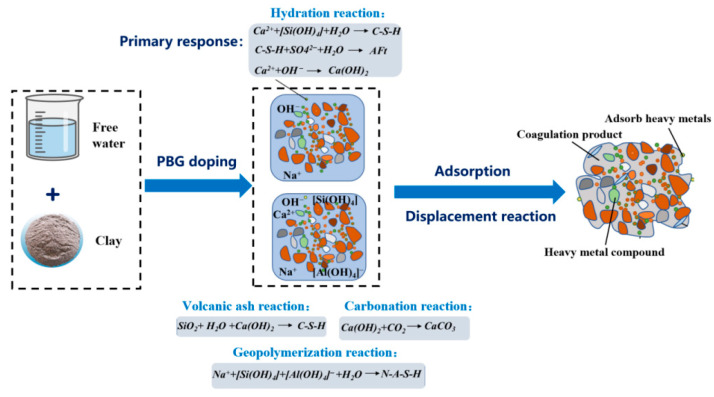
Phosphogypsum-based fluidized solidified soil reaction system.

In this section, SEM was employed to investigate the microstructure of PFSS and identify relevant microscopic patterns. SEM scans of the samples are presented in Figure 21, while the reaction system of PFSS is illustrated in Figure 22.

From Figure 21a, it can be observed that the amount of flocculent C-A-S-H gel product in PFSS is limited when the geopolymer dosage is 12%. This results in looser soil particles, leading to poorer mechanical properties and a reduced capacity to immobilize toxic substances. In Figure 21b, an increase in the quantity of C-A-S-H and C-S-H gels among the hydration products is evident, along with small clusters of AFt, suggesting that gel product formation improves with higher geopolymer dosages. Figure 21c,d show a substantial amount of C-A-S-H, C-S-H, and AFt gel products coating the surfaces of the soil particles, which further increases as geopolymer dosage rises. The AFt exhibits a needle-and-rod shape, allowing it to interpenetrate with C-A-S-H and C-S-H gels, forming a three-dimensional mesh structure. This mesh structure results in a denser matrix, enhancing mechanical strength and aiding in the encapsulation of toxic ions.

From Figure 22, it can be observed that hydration primarily contributes to mechanical strength, while carbonation, pozzolanic reactions, and geopolymerization serve as supplementary processes [26,36]. On the one hand, a large amount of Ca^2+^ dissolved under alkaline conditions can engage in displacement reactions with metal ions (such as Na^+^ and K^+^) on the surfaces of soil particles. This generates insoluble precipitates that react with toxic ions, including F^−^ and PO_4_^3−^. On the other hand, OH^−^ in the reaction system forms hydroxide precipitates with heavy metal cations, including As^5+^, Pb^2+^, and Cr^3+^. The hydrated gel products subsequently adsorb these precipitates, facilitating strength development and immobilization of toxic ions [37]. Additionally, Ca(OH)_2_ precipitates can carbonate with CO_2_ dissolved in water, producing CaCO_3_. This reduces the dispersion of soil particles, further enhancing the soil’s overall strength.

## 4. Conclusions

(1) The optimal formulation for phosphogypsum-based fluidized solidified soil (PFSS) was established, incorporating a geopolymer binder (composed of phosphogypsum and slag powder at 7:3, with alkali activator modulus of 1.4 and dosage of 14%) and a water-to-solid ratio between 0.48 and 0.50. The resulting PFSS exhibits favorable workability, with fluidity of 160–220 mm, initial and final setting times ≥ 3 h and ≤24 h, respectively, and a bleeding rate below 5%.
(2) PFSS achieves a 28-day compressive strength over 0.8 MPa, a California Bearing Ratio (CBR) above 8%, and a resilient modulus exceeding 40 MPa, thereby satisfying the mechanical requirements for backfilling in highway engineering, especially in poorly accessible zones. Leaching tests confirm that toxic ion concentrations are significantly reduced and comply with Groundwater Quality Standard III.
(3) The development of strength in PFSS is attributed to a combination of geopolymerization, pozzolanic reaction, ion exchange, and carbonation, with geopolymerization serving as the dominant mechanism. Toxic ions are effectively immobilized through physical encapsulation and chemical bonding within the hydrated gel matrix and AFt crystalline frameworks.
(4) While this study demonstrates the viability of PFSS for backfilling applications, certain limitations remain. The moderate strength and unvalidated long-term durability under field conditions warrant further investigation. Future work should focus on mix optimization for higher strength, durability testing under realistic environments, and holistic sustainability assessment.

## Figures and Tables

**Table 1:** Chemical composition of phosphogypsum (%).

Composition	Calcium Oxide	Sulfur Trioxide	Silicon Dioxide	Iron Oxide	Aluminum Oxide	Phosphorus Pentoxide	Strontium Oxide	Titanium Dioxide	Potassium Oxide	Sodium Oxide	Fluorine
Formula	CaO	SO_3_	SiO_2_	Fe_2_O_3_	Al_2_O_3_	P_2_O_5_	SrO	TiO_2_	K_2_O	Na_2_O	F
Content (%)	49.57	38.74	4.22	2.15	1.86	1.80	0.71	0.21	0.12	0.15	0.11

**Table 2:** Toxic substance leaching concentrations from phosphogypsum.

Toxic Substance	As (μg/L)	Pb (μg/L)	Cr (μg/L)	F^−^ (mg/L)	PO_4_^3−^ (mg/L)
Results	21.43	101.85	682.50	14.64	7.89

**Table 3:** Chemical composition of slag powder.

Composition	CaO	SiO_2_	Al_2_O_3_	MgO	SO_3_	TiO_2_	Na_2_O	MnO	Fe_2_O_3_	K_2_O
Content (%)	44.36	28.11	13.44	7.95	2.13	1.56	0.55	0.54	0.42	0.39

**Table 4:** Physical properties of soil.

Water Content (%)	Liquid Limit (%)	Plastic Limit (%)	Plasticity Index	Density (g/cm^3^)
7.6	42.8	23.6	19.2	1.91

**Table 5:** The ratio and strength of geopolymer.

Phosphogypsum:Slag Powder	Alkali Exciter Modulus	Dosage of Alkali Exciters	Water–Solid Ratios	Compressive Strength (MPa)		
				3 d	7 d	28 d
7:3	1.4	14%	0.52	9.34	10.67	11.41

**Table 6:** The strength requirements and proportioning ranges of geopolymer.

Usage Scenarios	Strength Requirements for Cementitious Materials (MPa)	Phosphogypsum (%)	Slag Powder (%)	Alkali Exciter (%)	Water–Solid Ratios	Compound Alkali Exciter Ratio		Sodium Methylsilicate (%)
						Modulus	NaOH/Na_2_SiO_3_	
Road backfilling	≥8.0	40~70	30~60	10~16	0.48~0.54	1.0~1.6	0.55~0.24	1.0

## Data Availability

The original contributions presented in this study are included in the article. Further inquiries can be directed to the corresponding authors.

## References

[1] Li C.A., Dong Y.G., Yi Y., Tian J., Xuan C., Wang Y., Wen Y., Cao J. (2023). Effects of phosphogypsum on enzyme activity and microbial community in acid soil. Sci. Rep..

[2] Ou L., Li R., Zhu H., Zhao H., Chen R. (2023). Upcycling waste phosphogypsum as an alternative filler for asphalt pavement. J. Clean. Prod..

[3] Ou Z.B., Yang W.J., He B.B. (2021). Current status of comprehensive utilisation of phosphogypsum at home and abroad. Yunnan Chem. Ind..

[4] He X.Y., Lv F., He G.Z. (2021). Pretreatment and comprehensive utilisation of industrial solid waste phosphogypsum in China. Resour. Conserv. Environ. Prot..

[5] Liu X.W., Zhang Y., Cheng M.J., Jiang S., Yuan Z. (2023). Recycling phosphorus from waste in China: Recycling methods and their environmental and resource consequences. Resour. Conversat. Recycl..

[6] Li B., Li L., Chen X., Ma Y., Zhou M. (2022). Modification of phosphogypsum using circulating fluidized bed fly ash and carbide slag for use as cement retarder. Constr. Build. Mater..

[7] Calderón-Morales B.R., García-Martínez A., Pineda P., García-Tenório R. (2021). Valorization of phosphogypsum in cement-based materials: Limits and potential in eco-efficient construction. J. Build. Eng..

[8] Lu G., Feng Z., Xu Y., Jin Y., Zhang G., Hu J., Yu T., Wang M., Liu M., Yang H. (2023). Impact of Phosphogypsum Application on Fungal Community Structure and Soil Health in Saline–Alkali-Affected Paddy Fields. Agronomy.

[9] Weiksnar K.D., Townsend T.G. (2023). Enhancing the chemical performance of phosphogypsum as a road base material by blending with common aggregates. Resour. Conserv. Recycl..

[10] Pu S.Y., Zhu Z.D., Huo W.W. (2021). Evaluation of engineering properties and environmental effect of recycled gypsum stabilized soil in geotechnical engineering: A comprehensive review. Resour. Conversat. Recycl..

[11] Değirmenci N. (2008). Utilization of phosphogypsum as raw and calcined material in manufacturing of building products. Constr. Build. Mater..

[12] Ngo H.T.T., Dang V.Q., Ho L.S., Doan T.X. (2022). Utilization phosphogypsum as a construction material for road base: A case study in Vietnam. Innov. Infrastruct. Solut..

[13] Liang Y., Guan B., Cao T., Liu G., Tang P., He M., Cheira M.F., Rashad A.M. (2023). Study on the properties of an excess-sulphate phosphogypsum slag cement stabilized base-course mixture containing phosphogypsum-based artificial aggregate. Constr. Build. Mater..

[14] Davidovits J. (2005). Geopolymers and geopolymeric materials. J. Therm. Anal. Calorim..

[15] Oubaha S., El Machi A., Mabroum S., Taha Y., Benzaazoua M., Hakkou R. (2024). Recycling of phosphogypsum and clay by-products from phosphate mines for sustainable alkali-activated construction materials. Constr. Build. Mater..

[16] Pratap B., Mondal S., Rao B.H. (2024). Mechanical and durability assessment of phosphogypsum- bauxite residue—Fly ash-based alkali-activated concrete. Constr. Build. Mater..

[17] Zhang Y., Ma Z., Zhi X., Chen X., Zhou J., Wei L., Liu Z. (2023). Damage characteristics and constitutive model of phosphogypsum/fly ash/slag recycled aggregate concrete under uniaxial compression. Cem. Concr. Compos..

[18] Zhao Y., Zhang N., Chen X. (2023). Test study on mechanical properties of compound municipal solid waste incinerator bottom ash premixed fluidized solidified soil. iScience.

[19] Tanabe L.K., Carvalho S., Dasari V., Nasif A., O’toole K.A., Berumen M.L. (2022). Potential effects of heavy metal pollution from a cement factory near Saudi Arabia’s largest green turtle rookery. Environ. Monit. Assess..

[20] (2017). Stander for Groundwater Quality. National Standards of PRC.

[21] Komljenović M., Baščarević Z., Bradić V. (2010). Mechanical and microstructural properties of alkali-activated fly ash geopolymers. J. Hazard. Mater..

[22] Lee L.T. (2004). Method to Rapidly Assess the Index Properties of Fine-Grained Dredged Materials. Geotech. Test J..

[23] (2002). Standard for Test Method of Performance on Ordinary Fresh Concrete. Building Materials Industry Standards of PRC.

[24] (2020). Test Methods of Cement and Concrete for Highway Engineering. Industry Standards of PRC.

[25] (2020). Test Methods of Soils for Highway Engineering. Industry Standards of PRC.

[26] Ji X.P., Liu J., Dong X.Z., Zhu S.Y., Li X.J. (2023). Preparation and Properties of Fly-ash-based Geopolymer for Stabilizing Tailing Subgrade Fille. China J. Highw. Transp..

[27] (2015). Technical Guidelines for Construction of Highway Roadbases. Industry Recommended Line Standards of PRC.

[28] (2022). Technical Standard for Engineering Application by Using Premixed Fluided Stabilized Soil. Local Standards for Engineering Construction in Sichuan Province, PRC.

[29] (2004). Specifications for Design of Highway Subgrades. Industry Standards of PRC.

[30] Rasaki S.A., Bingxue Z., Guarecuco R., Thomas T., Minghui Y. (2019). Geopolymer for use in heavy metals adsorption, and advanced oxidative processes: A critical review. J. Clean. Prod..

[31] Xu J., Chen P., Zhang C., Yang Y. (2023). Influence of phosphogypsum on mechanical properties and microstructure of iron tailings cementitious material. Arch. Civ. Mech. Eng..

[32] Weiksnar K.D., Clavier K.A., Laux S.J., Townsend T.G. (2023). Influence of trace chemical constituents in phosphogypsum for road base applications: A review. Resour. Conserv. Recycl..

[33] Eliwa A.A., Mubark A.E., Abdelfattah N.A., El Gawad E.A. (2022). Maximizing the exploitation of phosphogypsum wastes using soaking technique with citric acid, recovering rare-earth and residual phosphate contents. J. Cent. South Univ..

[34] Ji X., Chen B., Dong X., Lu H., Zhang X., He S., Wu T. (2023). Mechanical and environmental properties of geopolymer-stabilized domestic waste incineration slag in an asphalt pavement base. J. Road Eng..

[35] Nana A., Ngouné J., Kaze R.C., Boubakar L., Tchounang S.K., Tchakouté H.K., Kamseu E., Leonelli C. (2019). Room-temperature alkaline activation of feldspathic solid solutions: Development of high strength geopolymers. Constr. Build. Mater..

[36] Ji X.P., Dai C., Cui Z.F., Zhou R.Z. (2021). Research on engineering characteristics of curing agent stabilized phosphogypsum roadbed Filler. China J. Highw. Transp..

[37] Wu J., Xu T., Chu H., Xi X., Zhang F., Jin W. (2023). Study on Synergistic Effect of Xanthan Gum and Sodium Methylsiliconate on Mechanical Strength and Water Stability of Phosphogypsum Road-Based Materials. Materials.

